# Cumulative intra-abdominal pressure exposure and dynamic trajectories in ICU-admitted patients reveal prognostic determinants of severe acute pancreatitis

**DOI:** 10.1186/s13017-025-00646-y

**Published:** 2025-09-29

**Authors:** Maobin Kuang, Yaoyu Zou, Shixuan Xiong, Cong He, Nianshuang Li, Ling Ding, Xueyang Li, Huijie Zhang, Yupeng Lei, Xin Huang, Huifang Xiong, Lingyu Luo, Liang Xia, Wenhua He, Nonghua Lv, Jianhua Wan, Yin Zhu

**Affiliations:** 1 Jiangxi Provincial Key Laboratory of Digestive Diseases, Department of Gastroenterology, Jiangxi Clinical Research Center for Gastroenterology, Digestive Disease Hospital, The First Affiliated Hospital, Jiangxi Medical College, Nanchang University, No. 17 Yong Wai Zheng Street, Nanchang, 330006 Jiangxi China; 2 The First Clinical Medical College of Nanchang University, Nanchang, Jiangxi China

**Keywords:** Intra-abdominal pressure, SAP, CumIAP, IAP trajectory, Cumulative exposure, LCGMM

## Abstract

**Background:**

Intra-abdominal pressure (IAP) critically drives organ failure progression in severe acute pancreatitis (SAP). However, traditional static IAP monitoring inadequately captures dynamic injury evolution. This study aimed to assess the impact of cumulative IAP exposure (CumIAP) and dynamic trajectories on the prognosis of SAP.

**Methods:**

This retrospective cohort study analyzed 1,008 ICU-admitted SAP patients from the Jiangxi cohort (2005–2023) and 83 from MIMIC-IV (2008–2019). CumIAP was quantified via time-weighted integration of serial IAP measurements. Multivariate Cox regression models and restricted cubic splines (RCS) were established to analyze the dose–response relationships between CumIAP and death, infectious pancreatic necrosis (IPN), and persistent multiple organ failure (PMOF). Mediation analysis evaluated CumIAP’s role in albumin (ALB)- and acute necrotic collection (ANC)-associated outcomes. Latent class growth mixture model (LCGMM) was employed to identify dynamic IAP trajectory subtypes, and the associations between each trajectory group and poor prognosis were analyzed.

**Results:**

Over a median inpatient follow-up of 17 days in the Jiangxi cohort, 200 (19.8%) patients died in hospital, while 208 (20.6%) and 329 (32.6%) developed IPN and PMOF, respectively. Regression analysis revealed that for each standard deviation increase in CumIAP, the risks of in-hospital death and PMOF increased by 37% and 86%, respectively, and there was a U-shaped association with the risk of IPN (*P* for nonlinearity = 0.004). Mediation analysis showed that CumIAP mediated 24.26% and 33.76% of the associations between ALB, ANC, and the risk of in-hospital death, respectively. Three IAP trajectories were identified by LCGMM: the high-pressure rapid decline group (HRD-T1), the low-pressure gradual decline group (LGD-T2), and the low-pressure progressive increase group (LPI-T3). Among them, compared with HRD-T1 and LGD-T2, the subjects in the LPI-T3 group had a significantly increased risk of adverse clinical outcomes.

**Conclusion:**

This is the first study to revealed that CumIAP is linearly positively correlated with death and PMOF, while exhibits a U-shaped relationship with IPN. Notably, patients with low baseline IAP and a rising trajectory exhibited worse outcomes than those with high baseline IAP and a declining trend.

**Supplementary Information:**

The online version contains supplementary material available at 10.1186/s13017-025-00646-y.

## Highlights

This is the first study to introduce CumIAP as a quantitative metric to assess the early IAP burden and its prognostic significance in SAP, overcoming the limitations of static IAP monitoring.

RCS and regression analyses revealed a positive linear association between CumIAP and in hospital mortality/PMOF, and a U-shaped relationship with IPN, suggesting the possible presence of a risk threshold.

LCGMM identified three IAP trajectory patterns; the "low baseline with rising trend" group (LPI-T3) had the worst outcomes, underscoring the value of dynamic over static IAP monitoring.

Mediation analysis showed that CumIAP partly mediated the effects of hypoalbuminemia and ANC on outcomes, suggesting that IAP might represent an important intermediary pathway and a therapeutic target in SAP.

**Supplementary Information:**

The online version contains supplementary material available at 10.1186/s13017-025-00646-y.

## Background

Severe acute pancreatitis (SAP), a life-threatening abdominal emergency commonly encountered in intensive care units (ICUs), is characterized by an unpredictable disease course and high mortality rates (39–54%) [1–3]. The high mortality in SAP is largely attributed to its biphasic progression: an early phase of systemic inflammation-induced organ dysfunction, followed by sepsis from infected necrosis. This dynamic process leads to secondary organ failure in 40–50% of patients [2, 4–6], complicating timely intervention and limiting the effectiveness of conventional scoring systems (e.g., APACHE II, SOFA), which depend on static parameters and may miss evolving dysfunction.

Intra-abdominal pressure (IAP) monitoring provides crucial insights into SAP pathophysiology. Epidemiological data indicate that patients with SAP exhibit high incidence rates (61–84%) of intra-abdominal hypertension (IAH; defined as IAP ≥ 12 mmHg) and abdominal compartment syndrome (ACS; IAP > 20 mmHg with organ failure) [7–9]. Elevated IAP induces multiorgan dysfunction through mechanical compression and microcirculatory impairment, with levels strongly correlating with Sequential Organ Failure Assessment scores [9–11]. When IAP exceeds 25–33 mmHg, mortality rises sharply to 50%, and persistent IAH independently predicts 28- and 90-day mortality [9, 12]. Although international guidelines recommend routine IAP monitoring in at-risk ICU patients [13], current clinical practice remains limited. Most existing studies emphasize static measurements (e.g., peak or baseline values), neglecting dynamic fluctuations and the cumulative impact of IAP on patient outcomes. Reintam Blaser et al. demonstrated that persistent IAH during the first two ICU weeks has greater prognostic value than single measurements [12], highlighting the importance of pressure exposure duration. Moreover, distinct IAP fluctuation patterns (e.g., progressive increase vs. abrupt decrease) may exert differentially effects on outcomes via mechanisms such as altered abdominal perfusion pressure or ischemia–reperfusion injury [14–16]; however, these dynamic changes remain unquantified. This study, for the first time, comprehensively investigates “cumulative intra-abdominal pressure (CumIAP)” exposure and “dynamic IAP trajectory phenotypes” in SAP patients based on a large cohort. The primary objectives are to quantify the dose–response relationship between time-weighted CumIAP and the progression of persistent multiple organ failure (PMOF), infected pancreatic necrosis (IPN), and mortality risk, and to identify high-risk IAP fluctuation patterns. This work aims to provide evidence supporting the shift from empirical management to a precision medicine approach guided by real-time dynamic data, ultimately improving patient outcomes and reducing SAP-related mortality.

## Methods

### Study design and data source

This study was a retrospective cohort analysis utilizing patient data obtained from the electronic medical database continuously maintained by the Department of Gastroenterology, the First Affiliated Hospital of Nanchang University (Jiangxi cohort, 2005–2023), and the Medical Information Mart for Intensive Care IV database (MIMIC-IV v2.2, 2008–2019). Detailed descriptions of both databases are provided in Supplementary Method 1.

### Study population and ethical approval

**Fig. 1:**
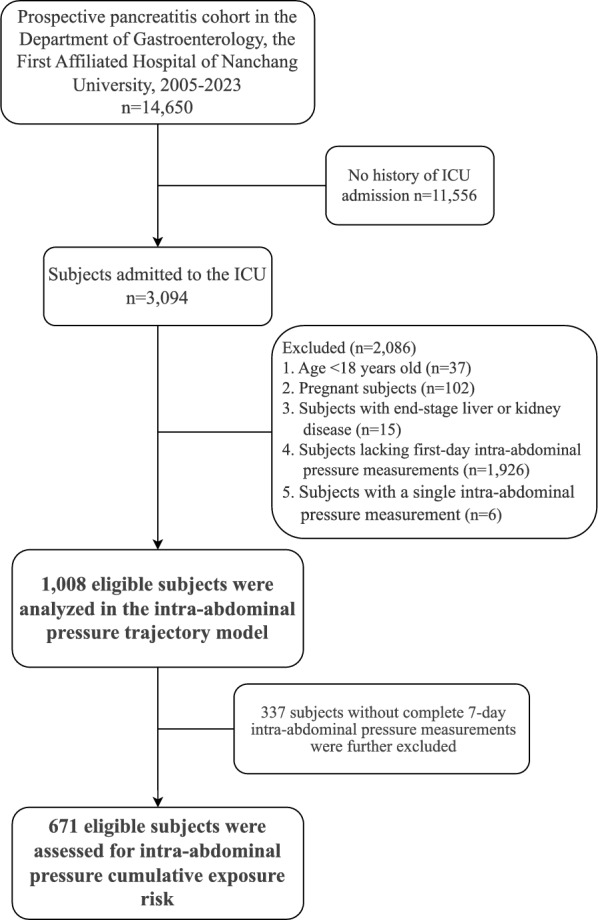
Flow chart of inclusion and exclusion of study subjects

From the Jiangxi cohort of 14,650 hospitalized AP patients, 3,094 ICU admissions were identified. After excluding patients under 18 years old, pregnant women, those with end-stage liver or kidney disease, missing baseline IAP data, or only a single IAP measurement, 1,008 patients were eligible for IAP trajectory analysis. To assess the impact of cumulative IAP burden within the first week of ICU stay, 337 patients lacking complete IAP records on days 1, 2, 3, 5, and 7 were further excluded, leaving 671 patients for CumIAP analysis (Fig. 1). Separately, 1280 AP-related ICU admissions were extracted from the MIMIC-IV v2.2 database. After excluding non-first admissions (n = 169), patients aged < 18 or > 75 years (n = 246), ICU stays < 24 h (n = 132), and those with only a single IAP record (n = 650), 83 patients remained for trajectory modeling. However, due to limited longitudinal IAP data (n = 18 with complete IAP records on days 1, 2, 3, 5, and 7), MIMIC-IV was not suitable for CumIAP analysis.

This study was approved by the Institutional Review Boards of the First Affiliated Hospital of Nanchang University (Approval No. 2011001) and the Massachusetts Institute of Technology. All procedures followed the Declaration of Helsinki and STROBE guidelines. (Supplementary Method 2) [17, 18].

### Data collection and missing data handling

Multidimensional data were extracted from the structured electronic medical database of the First Affiliated Hospital of Nanchang University, encompassing demographics and anthropometrics (age, gender, weight), smoking status, drinking status, etiology, comorbidities (history of hyperlipidemia and diabetes), vital signs [temperature, pulse, respiratory, systolic blood pressure (SBP), diastolic blood pressure (DBP)], laboratory test [white blood cell count (WBC), platelet count (PLT), hematocrit (HCT), total bilirubin (TBIL), albumin (ALB), triglyceride (TG), total cholesterol (TC), creatinine (Cr), blood urea nitrogen (BUN)], medications and treatments [Insulin, albumin supplementation, low-molecular-weight heparin (LMWH), surgical, percutaneous catheter drainage (PCD), volume of fluid resuscitation (VFR)], APACHE II, and in-hospital outcomes such as length of stay (LOS), acute necrotic collection (ANC), IPN, PMOF, and mortality. Data from the MIMIC-IV v2.2 database were retrieved using SQL via Navicat Premium 15.0.12, focusing on repeated IAP measurements during ICU stay and in-hospital mortality for trajectory modeling.

Missing data were assessed by calculating the proportion of missingness for each variable (Table S1), and appropriate imputation methods were applied based on missingness degree [19, 20], as detailed in Supplementary Method 3.

### Measurement of IAP and calculation of CumIAP

IAP was measured using the standardized bladder manometry method. Patients were placed in the supine position, 25 mL of sterile saline was instilled via a Foley catheter, and a pressure transducer was connected. The pubic symphysis was used as the zero reference point, and IAP was recorded at end-expiration [12, 21]. The worst IAP values on days 1, 2, 3, 5, and 7 after ICU admission were used to calculate the CumIAP over the first week. The calculation formula is CumIAP = (IAP_day1_ + IAP_day2_)/2 + (IAP_day2_ + IAP_day3_)/2 + (IAP_day3_ + IAP_day5_) + (IAP_day5_ + IAP_day7_) [22–24].

### Diagnosis of SAP and research outcomes

According to the Revised Atlanta Classification in 2012 [2], AP can be diagnosed if the following two items are met: (1) Typical symptoms (e.g., severe acute upper abdominal pain); (2) Serum amylase or lipase levels > 3 times the upper limit of the normal; (3) CT/MRI shows pancreatic inflammatory changes (such as enlargement, exudation, necrosis). On this basis, SAP is defined as the presence of persistent organ failure (≥ 48 h, modified Marshall score ≥ 2 points, such as respiratory failure, circulatory failure, acute renal failure, etc.).

The primary outcome of this study was in-hospital mortality. Secondary outcomes included IPN and PMOF during the hospital stay. Follow-up began at ICU admission and ended at either in-hospital death or discharge, whichever occurred first.

### Statistical analysis

**Descriptive analysis**: All subjects were grouped according to the tertiles of CumIAP to compare the baseline characteristics and clinical outcomes. Continuous variables were assessed for normality using the Kolmogorov–Smirnov test and Q-Q plots (Table S2, Fig. S1). Variables with normal distribution were presented as mean ± standard deviation (SD) and compared using one-way ANOVA, while non-normally distributed variables were expressed as median (interquartile range) and compared using the Kruskal–Wallis H test. Categorical variables were expressed as frequency (percentage), and the chi-square test was used for the comparison between groups. Kaplan–Meier curves were drawn and log-rank tests were performed to compare the differences in cumulative survival risks among the three groups of CumIAP.

**Correlation analysis**: Before the regression analysis, multicollinearity was assessed using multiple linear regression. Variables with a variance inflation factor value > 5 were considered to exhibit multicollinearity and were excluded from the multivariable regression models [25]. In addition, schoenfeld residual analysis was used to verify whether the association between CumIAP and the risk of in-hospital death conformed to the proportional hazards assumption. Cox regression was applied to evaluate the association between CumIAP and in-hospital mortality, while multivariate logistic regression assessed correlations with IPN and PMOF. Dose–response relationships between CumIAP and adverse outcomes were modeled using restricted cubic splines (RCS) with four knots. According to the results of the collinearity analysis and clinical relevance, in the above regression model, the demographic characteristic indicators (age, gender) of the subjects, past medical history (history of diabetes, history of hyperlipidemia), the four vital signs (temperature, pulse, respirations, SBP) upon ICU admission, and laboratory test indicators (ALB, TC, Cr, HCT, PLT) were adjusted. Additionally, the predictive performance of CumIAP for adverse outcomes was assessed using receiver operating characteristic curves, with calculation of the area under the curve (AUC), optimal cutoff values, sensitivity, and specificity.

**Mediation effect analysis**: To deeply analyze the potential pathological mechanism and clinical significance of CumIAP, a mediation effect analysis was further carried out. The total effect and direct effect of the association between ALB, ANC and the risks of in-hospital death, IPN, and PMOF were calculated, as well as the indirect effect of CumIAP. The mediation percentage was calculated to evaluate the mediating role of CumIAP between ALB, ANC and the poor prognosis of the subjects. The same variables as in the above regression model were adjusted in the mediation analysis model.

**Subgroup and sensitivity analysis**: Stratified analysis was conducted according to gender, age (60 years old), drinking status, smoking status, and the presence of comorbidities (diabetes, hyperlipidemia), with interaction tested using likelihood ratio tests. In addition, five sensitivity analyses were performed, with the specific methods detailed in Supplementary Method 4.

**Analysis of latent class growth mixture model (LCGMM)**: To compensate for the lack of dynamic fluctuation information in CumIAP, LCGMM analysis was conducted on patients who had at least two IAP measurements within the first 7 days after ICU admission. Maximum likelihood estimation was used to model the IAP trajectories over this 7-day period. Details of the LCGMM construction are provided in Supplementary Method 5 [26–29]. Subsequently, baseline and outcome differences were compared across trajectory groups, and their prognostic value was further assessed using Cox and logistic regression. An external validation was performed using SAP patients in the MIMIC-IV cohort (n = 83), with group distributions and outcomes compared accordingly.

All statistical analyses of this study were completed using R 4.2.2, Free Statistics 2.3, and Empower 2.0, and a two-sided *P* < 0.05 was set as statistically significant.

## Results

### Baseline characteristics of subjects

**Fig. 2:**
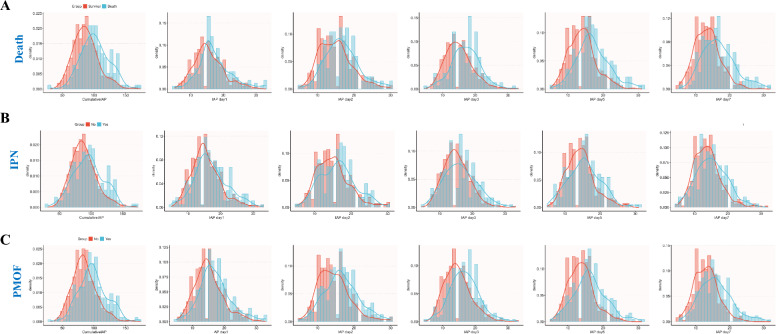
Grouped Presentation of CumIAP Exposure and IAP Levels on Days 1, 2, 3, 5, and 7 in Subjects with Death (**A**), IPN (**B**), and PMOF (**C**) outcome, respectively. CumIAP: Cumulative Intra-abdominal Pressure; IAP: Intra-abdominal Pressure; IPN: Infected Pancreatic Necrosis; PMOF: Persistent Multiple Organ Failure

**Fig. 3:**
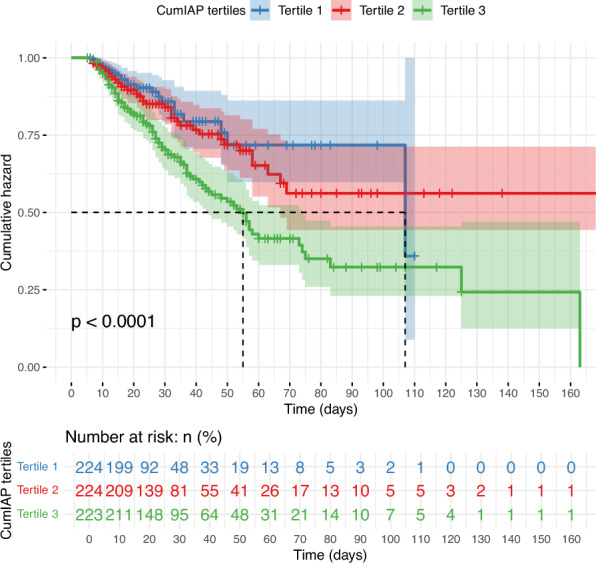
The Kaplan–Meier Survival Curves of Subjects Grouped by CumIAP Tertiles. CumIAP: Cumulative Intra-abdominal Pressure

A total of 671 SAP patients who completed 7-day IAP monitoring was included in this study for cumulative exposure analysis. Among them, there were 259 (38.6%) females and 412 (61.4%) males, with a median age of 50.2 years. After grouping the subjects by CumIAP tertiles, significant differences were observed in their baseline characteristics and clinical outcomes (Table 1). Specifically, compared with the T1 group, the T3 group had higher baseline IAP, Cr, BUN, TG, APACHE II scores, larger VFR, and higher rates of surgical or PCD interventions (all *P* < 0.05). In terms of etiologies, biliary pancreatitis predominated in T1 (52.2%), whereas hypertriglyceridemia-induced pancreatitis was most frequent in T3 (47.8%, *P* < 0.001). In addition, the probability density curve and histogram showed that CumIAP had better discrimination for clinical outcomes (Death, IPN, and PMOF) compared with the IAP measured on a single day (Day 1/2/3/5/7) (Fig. 2). In terms of clinical outcomes, the LOS, ANC, IPN, and PMOF in the T3 group were significantly higher (All *P* < 0.001) (Table 2). Moreover, Kaplan–Meier analysis showed significantly higher mortality in T3 (39.3% vs. 11.2% in T1 and 19.3% in T2; Log-rank *P* < 0.05) (Fig. 3).

**Table 1:** Baseline characteristics of subjects grouped according to CumIAP tertiles

Variables	Total (n = 671)	Tertile 1 (n = 224)	Tertile 2 (n = 223)	Tertile 3 (n = 224)	*P*
*Demographic features*					
Female, n (%)	259 (38.6)	104 (46.4)	90 (40.4)	65 (29)	< 0.001
Age, year	49.0 (38.0, 62.0)	50.5 (41.0, 64.2)	48.0 (37.0, 62.5)	47.5 (38.0, 58.2)	0.126
Weight, kg	67.0 (60.0, 75.0)	63.8 (56.0, 70.0)	67.0 (60.0, 75.0)	70.0 (62.4, 80.0)	< 0.001
Smoking, n (%)	222 (33.1)	68 (30.4)	77 (34.5)	77 (34.4)	0.568
Drinking, n (%)	245 (36.5)	71 (31.7)	76 (34.1)	98 (43.8)	0.02
*Etiology and comorbidities*					
Etiology, n (%)					< 0.001
Biliary	288 (42.9)	117 (52.2)	100 (44.8)	71 (31.7)	
HTG	240 (35.8)	55 (24.6)	78 (35)	107 (47.8)	
Alcoholic	51 (7.6)	19 (8.5)	15 (6.7)	17 (7.6)	
Others	92 (13.7)	33 (14.7)	30 (13.5)	29 (12.9)	
Hyperlipidemia, n (%)	88 (13.1)	27 (12.1)	26 (11.7)	35 (15.6)	0.392
Diabetes, n (%)	99 (14.8)	34 (15.2)	32 (14.3)	33 (14.7)	0.97
*Vital signs*					
Temperature, ℃	37.2 (36.7, 38.0)	37.2 (36.7, 37.9)	37.2 (36.7, 37.9)	37.3 (36.8, 38.0)	0.344
Pulse, times/min	110.0 (98.0, 125.0)	108.0 (94.0, 122.0)	111.0 (100.0, 124.0)	116.0 (98.0, 128.0)	0.024
Respirations, times/min	27.0 (22.0, 34.0)	26.0 (22.0, 32.0)	28.0 (22.0, 34.0)	28.5 (22.0, 34.2)	0.108
SBP, mmHg	131.0 (117.0, 147.0)	131.5 (117.0, 146.2)	132.0 (117.0, 146.5)	131.0 (118.0, 147.0)	0.975
DBP, mmHg	82.0 (72.0, 92.0)	81.0 (72.0, 90.2)	83.0 (72.5, 93.5)	83.0 (73.0, 93.0)	0.578
*Laboratory tests*					
IAP, mmHg	15.0 (13.0, 18.6)	12.0 (10.0, 14.0)	15.0 (14.0, 17.0)	19.0 (17.0, 23.0)	< 0.001
TBIL, umol/L	22.3 (14.1, 36.8)	20.4 (14.0, 34.5)	22.8 (13.8, 37.3)	23.1 (14.7, 36.7)	0.565
Cr, umol/L	87.9 (59.4, 175.0)	76.3 (55.2, 111.6)	91.4 (62.9, 182.0)	99.6 (63.8, 219.9)	< 0.001
CRP, mg/L	191.4 (101.5, 275.5)	181.0 (38.1, 284.1)	182.9 (110.0, 238.9)	209.7 (120.2, 303.9)	0.267
ALB, g/L	33.0 (30.0, 36.8)	33.4 (30.3, 37.9)	32.7 (29.7, 36.3)	32.7 (30.0, 36.3)	0.287
TC, mmol/L	4.2 (3.2, 6.9)	4.1 (3.2, 6.5)	4.1 (3.0, 6.4)	4.5 (3.3, 9.1)	0.164
TG, mmol/L	2.5 (1.3, 8.6)	2.1 (1.2, 5.5)	2.4 (1.2, 6.8)	4.7 (1.5, 13.4)	< 0.001
APACHE II	12.0 (9.0, 16.0)	11.0 (8.0, 15.0)	13.0 (8.0, 16.0)	13.0 (10.0, 16.0)	0.014
BUN, mmol/L	7.5 (4.9, 11.5)	6.6 (4.7, 10.0)	7.7 (4.8, 11.4)	8.2 (5.2, 13.2)	0.006
WBC, 10^9^/L	13.3 (10.0, 18.0)	14.2 (10.0, 19.0)	13.4 (10.4, 17.5)	13.0 (10.0, 18.3)	0.659
PLT, × 10^9^/L	184.5 (134.0, 241.0)	189.0 (140.0, 245.0)	183.0 (130.0, 238.5)	178.0 (128.8, 234.0)	0.284
HCT, n (%)	40.8 (33.3, 47.0)	40.0 (33.7, 45.4)	40.7 (33.0, 48.1)	41.4 (33.3, 47.1)	0.368
*Medications and treatments*					
Insulin, n (%)	597 (89.0)	187 (83.5)	206 (92.4)	204 (91.1)	0.005
Alb Suppl, n (%)	553 (82.4)	169 (75.4)	188 (84.3)	196 (87.5)	0.002
LMWH, n (%)	251 (37.4)	75 (33.5)	88 (39.5)	88 (39.3)	0.331
Surgical, n (%)	24 (3.6)	4 (1.8)	6 (2.7)	14 (6.2)	0.027
PCD, n (%)	225 (33.5)	64 (28.6)	63 (28.3)	98 (43.8)	< 0.001
VFR, ml	2800.0 (2095.0, 3745.0)	2587. 0(1989.5, 3456.0)	2730.0 (2050.5, 3640.0)	3146.0 (2357.5, 4231.5)	< 0.001

IAP, Intra-abdominal Pressure; CumIAP, Cumulative Intra-abdominal Pressure; SBP, Systolic Blood Pressure; DBP, Diastolic Blood Pressure; TBIL, Total Bilirubin; Cr, Creatinine; CRP, C-Reactive Protein; ALB, Albumin; TC, Total Cholesterol; TG, Triglyceride; APACHEII, Acute Physiology and Chronic Health Evaluation II; BUN, Blood Urea Nitrogen; WBC, White Blood Cell; NEU, Neutrophil; PLT, Platelet; HCT, Hematocrit; HTG, Hypertriglyceridemia; Alb Suppl, Albumin Supplementation; LMWH, Low-Molecular-Weight Heparin; PCD, Percutaneous Catheter Drainage; VFR, Volume of Fluid Resuscitation

**Table 2:** Clinical outcomes of subjects grouped according to CumIAP Tertiles

	Total (n = 671)	Tertile 1 (n = 224)	Tertile 2 (n = 223)	Tertile 3 (n = 223)	*P*
LOS (days)	22.0 (15.0, 37.0)	18.0 (13.0, 28.0)	22.0 (15.0, 38.0)	26.0 (16.0, 44.0)	< 0.001
ANC, n (%)					< 0.001
No	237 (35.3)	106 (47.3)	68 (30.5)	63 (28.1)	
Yes	434 (64.7)	118 (52.7)	155 (69.5)	161 (71.9)	
IPN, n (%)					< 0.001
No	487 (72.6)	177 (79)	172 (77.1)	138 (61.6)	
Yes	184 (27.4)	47 (21)	51 (22.9)	86 (38.4)	
PMOF, n (%)					< 0.001
No	397 (59.2)	168 (75)	140 (62.8)	89 (39.7)	
Yes	274 (40.8)	56 (25)	83 (37.2)	135 (60.3)	
Death, n (%)					< 0.001
No	515 (76.8)	199 (88.8)	180 (80.7)	136 (60.7)	
Yes	156 (23.2)	25 (11.2)	43 (19.3)	88 (39.3)	

IAP, Intra-abdominal Pressure; CumIAP, Cumulative Intra-abdominal Pressure; LOS, Length of Hospital Stay; ANC, Acute Necrotic Collection; IPN, Infected Pancreatic Necrosis; PMOF, Persistent Multiple Organ Failure

### Association between CumIAP and poor prognosis of SAP subjects

**Fig. 4:**
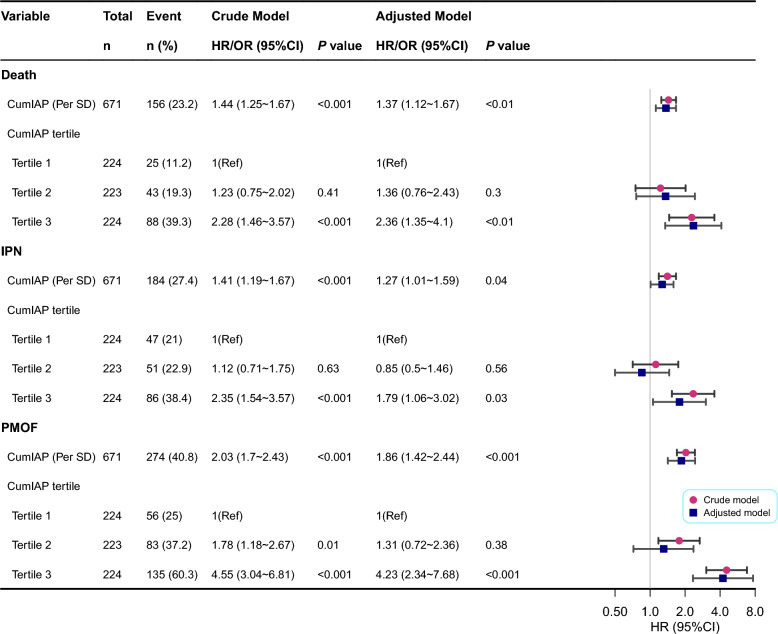
Association Analysis Between CumIAP and the Risk of Death, IPN, and PMOF. CumIAP: Cumulative Intra-abdominal Pressure; IPN: infected pancreatic necrosis; PMOF: persistent multiple organ failure. Crede Model was adjusted for: None. Adjusted Model: Adjusted for Sex, Age, Temperature, Pulse, Respirations, SBP, History of diabetes, History of hyperlipidemia, ALB, TC, Cr, HCT, PLT

**Fig. 5:**
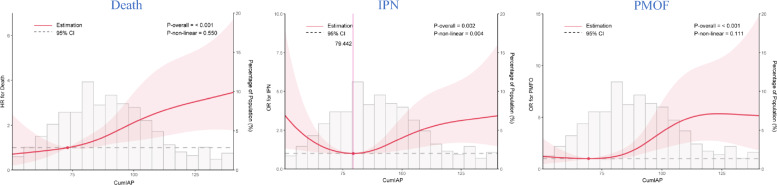
Restricted Cubic Spline Regression Analysis between CumIAP and the Risk of Death, SAP, IPN, and PMOF, respectively. CumIAP: Cumulative Intra-abdominal Pressure; IPN: Infected Pancreatic Necrosis; PMOF: Persistent Multiple Organ Failure

Prior to Cox regression, the proportional hazards assumption was confirmed and collinearity was assessed. CumIAP met the proportional hazards assumption, and no collinear variables were identified (Table S3 and Fig. S2). Subsequently, in multivariable Cox regression, CumIAP was significantly associated with adverse outcomes. Each SD increase in CumIAP was associated with a 37% higher risk of in-hospital mortality (HR = 1.37, 95% CI: 1.12–1.67), a 27% higher risk of IPN (HR = 1.27, 95% CI: 1.01–1.59), and an 86% higher risk of PMOF (HR = 1.86, 95% CI: 1.42–2.44). When CumIAP was analyzed categorically, patients in the T3 group had significantly higher risks of poor outcomes than T1 (Fig. 4). RCS models showed a positive linear relationship with mortality and PMOF, and a U-shaped relationship with IPN *(P* for nonlinearity = 0.004), with the lowest IPN risk observed at around 79.44 mmHg (Fig. 5). Further ROC analysis identified optimal CumIAP cutoffs of approximately 90.91 for predicting in-hospital mortality and PMOF, and 89.25 for predicting IPN, with AUCs of 0.70, 0.67, and 0.69, respectively (Fig. S3 and Table S4), indicating acceptable but not strong discrimination.

### Mediation effect analysis of CumIAP on the risks of poor prognosis of SAP associated with ALB and ANC

**Fig. 6:**
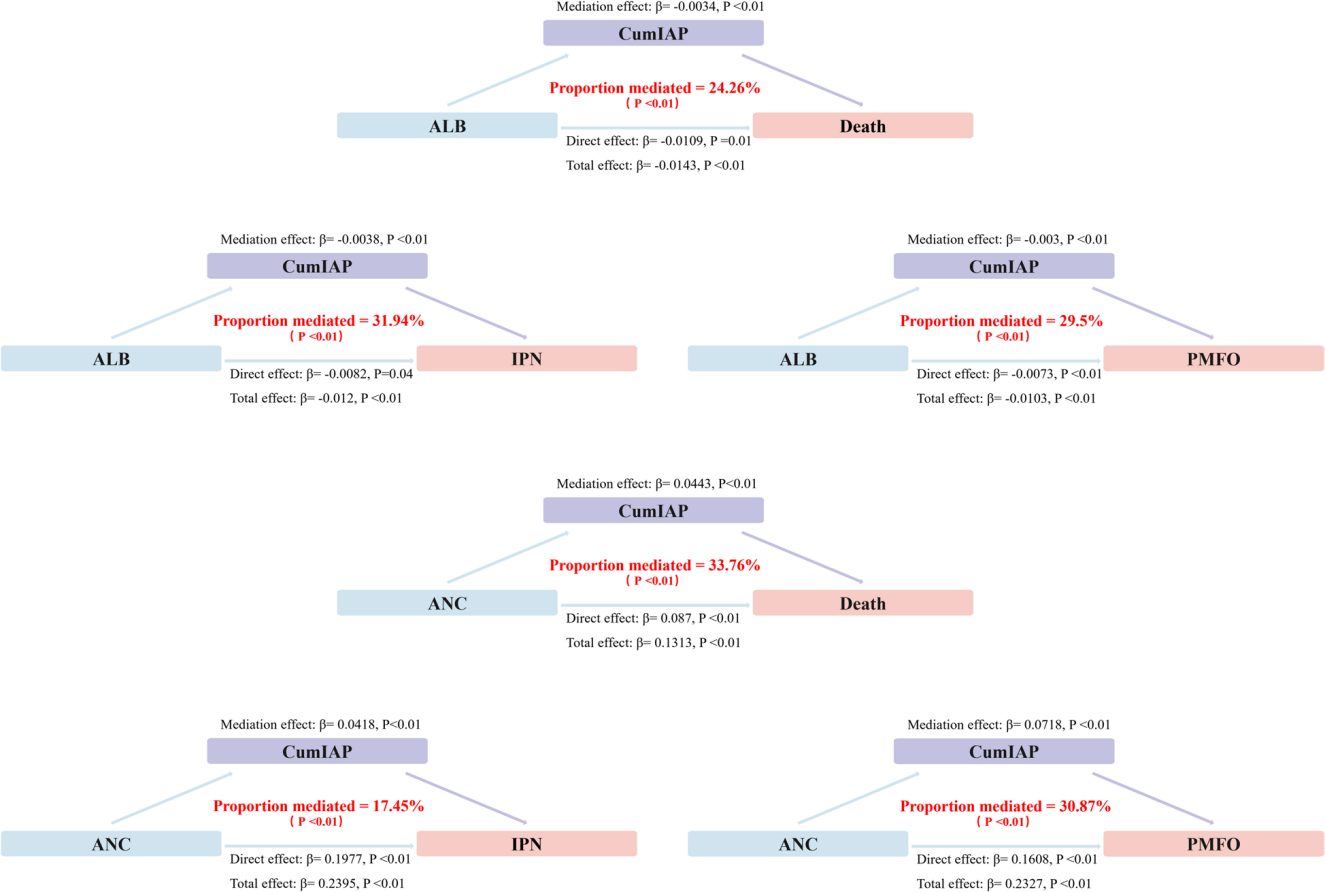
Mediation analysis of the role of CumIAP in the effects of ALB and ANC on the risk of adverse outcomes in acute pancreatitis. CumIAP, Cumulative Intra-abdominal Pressure; ALB, Albumin; ANC, Acute Necrotic Collection

Using CumIAP as a mediating variable, its role in the association between ALB, ANC and the poor prognosis of SAP patients was explored. The results showed that CumIAP partially mediated the associations of ALB and ANC with poor prognosis (Fig. 6). Specifically, for the ALB-related outcomes, the proportion of the mediation effect of CumIAP ranged from 24.26% (Death, β = -0.0034, *P* < 0.01) to 31.94% (IPN, β = -0.0038, *P* < 0.01), indicating that at least 24.6% of the reduced risk of poor prognosis by supplementing ALB could be explained by reducing CumIAP. For the ANC-related outcomes, CumIAP mediated 17.45% of the association with IPN, 30.87% with PMOF, and 33.76% with mortality, indicating that almost one-third of the death risk attributed to ANC was mediated through elevated CumIAP.

### Subgroup and sensitivity analysis of the risk of in-hospital death of subjects associated with CumIAP

Subgroup analysis stratified by sex, age, smoking and drinking status, and history of diabetes and hyperlipidemia (Fig. S4) revealed that the association between CumIAP and in-hospital mortality was stronger among females, patients with hyperlipidemia, and non-smokers. Five sensitivity analyses, including multiple imputation, use of cumulative vital signs, additional treatment adjustments, exclusion of elderly patients, and further adjustment for interventions (Tables S5–S6), yielded consistent results. Across all sensitivity analyses, the HR values of CumIAP ranged from 1.28 (1.05–1.58) to 1.45 (1.23–1.71), confirming a robust positive association between CumIAP and in-hospital mortality.

### Dynamic change trajectory of IAP of subjects and its association with poor prognosis

**Fig. 7:**
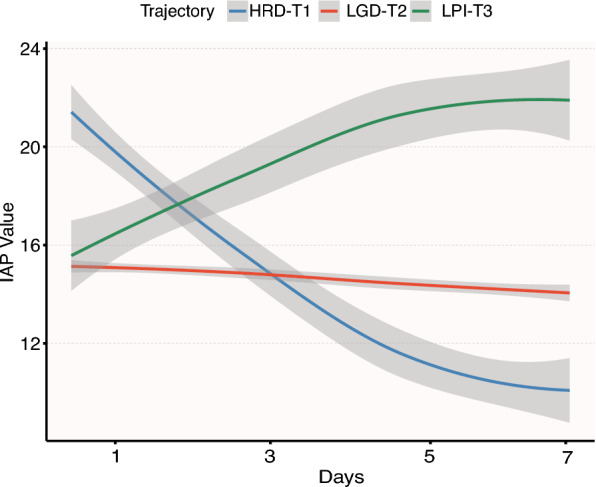
Potential Class Change Trajectories of IAP in Subjects form Jiangxi Cohort (n = 1,008). IAP, Intra-abdominal Pressure; LGD, Low-pressure Gradual Decline; HRD, High-pressure Rapid Decline; LPI, Low-pressure Progressive Increase

All LCGMMs were evaluated based on convergence (coefficient = 1), lowest Bayesian Information Criterion, highest entropy, and posterior probability (PP > 70%). The optimal model identified three distinct IAP trajectories (Table S7); (1) high-pressure rapid decline group (HRD-T1, n = 56); (2) low-pressure gradual decline group (LGD-T2, n = 909); (3) low-pressure progressive increase group (LPI-T3, n = 43) (Fig. 7). Compared with LGD-T2, LPI-T3 patients had higher Cr, BUN, APACHE II scores, and more interventions, while HRD-T1 had the highest baseline IAP (20 mmHg vs. 15 in LGD-T2 and 15.5 in LPI-T3; *P* < 0.001) (Table 3). HRD-T1 and LPI-T3 exhibited opposite IAP trajectories.

**Table 3:** Baseline Characteristics of Subjects Grouped According to IAP Trajectories

Variables	Total (n = 1,008)	HRD-T1 (n = 56)	LGD-T2 (n = 909)	LPI-T3 (n = 43)	*P*
*Demographic features*					
Female, n (%)	414 (41.1)	17 (30.4)	387 (42.6)	10 (23.3)	< 0.001
Age, year	50.0 (40.0, 64.0)	47.0 (38.8, 58.0)	51.0 (40.0, 65.0)	52.0 (44.5, 60.0)	0.439
Weight, kg	65.0 (59.0, 75.0)	70.0 (61.5, 80.0)	65.0 (58.0, 75.0)	70.0 (63.0, 75.0)	0.041
Smoking, n (%)	304 (30.2)	23 (41.1)	262 (28.8)	19 (44.2)	0.019
Drinking, n (%)	337 (33.4)	24 (42.9)	291 (32)	22 (51.2)	0.01
*Etiology and comorbidities*					
Etiology, n (%)					0.385
Biliary	467 (46.3)	22 (39.3)	428 (47.1)	17 (39.5)	
HTG	337 (33.4)	19 (33.9)	305 (33.6)	13 (30.2)	
Alcoholic	75 (7.4)	4 (7.1)	66 (7.3)	5 (11.6)	
Others	129 (12.8)	11 (19.6)	110 (12.1)	8 (18.6)	
Hyperlipidemia, n (%)	125 (12.4)	10 (17.9)	108 (11.9)	7 (16.3)	0.308
Diabetes, n (%)	143 (14.2)	5 (8.9)	134 (14.7)	4 (9.3)	0.31
*Vital signs*					
Temperature, ℃	37.2 (36.7, 37.9)	37.2 (36.6, 38.0)	37.2 (36.7, 37.9)	37.0 (36.7, 37.9)	0.686
Pulse, times/min	108.0 (92.0, 123.0)	106.0 (92.8, 125.8)	107.0 (92.0, 123.0)	112.0 (98.5, 123.0)	0.538
Respirations, times/min	26.0 (21.0, 33.0)	28.0 (22.8, 34.2)	26.0 (22.0, 33.0)	24.0 (20.0, 32.5)	0.257
SBP, mmHg	132.0 (117.0, 147.0)	129.0 (115.8, 145.0)	132.0 (117.0, 147.0)	133.0 (117.5, 150.0)	0.841
DBP, mmHg	82.0 (72.0, 92.0)	83.0 (71.0, 90.5)	82.0 (72.0, 92.0)	84.0 (72.0, 93.5)	0.893
*Laboratory tests*					
IAP, mmHg	15.0 (12.0, 18.0)	20.0 (17.8, 24.0)	15.0 (12.0, 18.0)	15.5 (12.5, 18.5)	< 0.001
TBIL, umol/L	20.2 (13.3, 35.7)	18.9 (12.7, 27.0)	20.1 (13.1, 36.4)	25.1 (15.3, 58.4)	0.06
Cr, umol/L	80.5 (57.7, 148.9)	87.3 (59.4, 115.0)	78.1 (57.0, 142.1)	178.1 (86.0, 332.5)	< 0.001
CRP, mg/L	187.2 (92.9, 274.9)	210.0 (169.0, 262.5)	182.9 (88.2, 270.0)	275.3 (155.8, 362.8)	0.265
ALB, g/L	33.5 (30.4, 37.9)	33.0 (31.0, 35.5)	33.7 (30.4, 38.0)	31.5 (27.9, 33.8)	0.094
TC, mmol/L	4.2 (3.2, 6.6)	4.5 (3.3, 7.9)	4.1 (3.2, 6.5)	4.5 (3.3, 7.7)	0.531
TG, mmol/L	2.1 (1.2, 7.7)	2.5 (1.3, 11.2)	2.1 (1.1, 7.2)	4.1 (1.5, 9.6)	0.123
APACHE II	12.0 (8.0, 15.0)	10.0 (7.0, 14.5)	12.0 (8.0, 15.0)	14.5 (10.0, 17.0)	0.008
BUN, mmol/L	6.9 (4.6, 11.0)	6.8 (4.6, 10.1)	6.8 (4.6, 10.6)	11.5 (7.2, 17.0)	< 0.001
WBC, 109/L	13.3 (10.0, 18.0)	14.3 (10.0, 18.0)	13.2 (10.0, 18.0)	14.0 (10.0, 20.0)	0.798
PLT, × 109/L	184.0 (132.0, 241.0)	198.0 (156.5, 231.0)	183.0 (133.0, 242.0)	168.5 (101.5, 244.2)	0.382
HCT, %	40.7 (34.0, 46.4)	43.0 (36.1, 48.9)	40.5 (34.1, 46.1)	40.5 (29.0, 46.9)	0.153
*Medications and treatments*					
Insulin, n (%)	852 (84.5)	51 (91.1)	762 (83.8)	39 (90.7)	0.18
Alb Suppl, n (%)	770 (76.4)	45 (80.4)	688 (75.7)	37 (86)	0.228
LMWH, n (%)	312 (31.0)	17 (30.4)	279 (30.7)	16 (37.2)	0.662
Surgical, n (%)	27 (2.7)	0 (0)	23 (2.5)	4 (9.3)	0.034
PCD, n (%)	284 (28.2)	26 (46.4)	245 (27)	13 (30.2)	0.007
VFR, ml	2750.0 (2050.0, 3705.0)	3200.0 (2217.5, 4814.0)	2740.0 (2045.0, 3668.5)	2561.0 (2100.0, 3774.0)	0.126

IAP, Intra-abdominal Pressure; CumIAP, Cumulative Intra-abdominal Pressure; SBP, Systolic Blood Pressure; DBP, Diastolic Blood Pressure; TBIL, Total Bilirubin; Cr, Creatinine; CRP, C-Reactive Protein; ALB, Albumin; TC, Total Cholesterol; TG, Triglyceride; APACHEII, Acute Physiology and Chronic Health Evaluation II; BUN, Blood Urea Nitrogen; WBC, White Blood Cell; PLT, Platelet; HCT, Hematocrit; HTG, Hypertriglyceridemia; Alb Suppl, Albumin Supplementation; LMWH, Low-Molecular-Weight Heparin; PCD, Percutaneous Catheter Drainage; VFR, Volume of Fluid Resuscitation

**Fig. 8:**
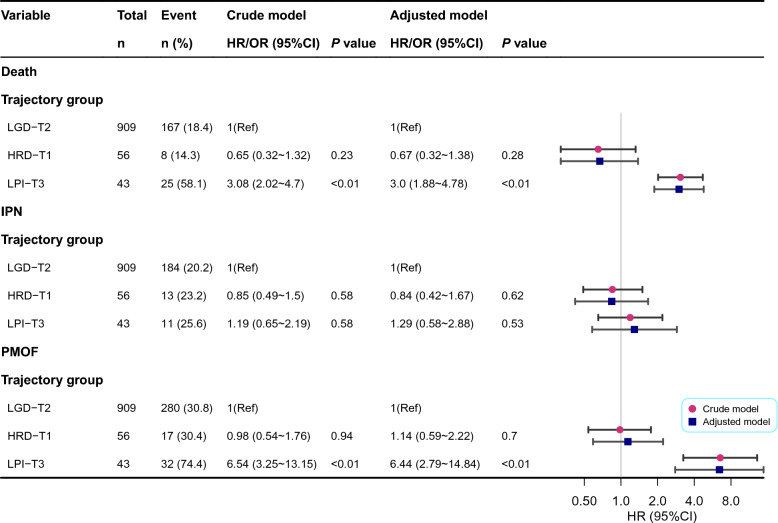
Association Analysis Between IAP trajectories and the Risk of Death, IPN, and PMOF. IAP, Intra-abdominal Pressure; IPN, infected pancreatic necrosis; PMOF, persistent multiple organ failure; LGD, Low-pressure Gradual Decline; HRD, High-pressure Rapid Decline; LPI, Low-pressure Progressive Increase. Crede Model was adjusted for: None. Adjusted Model: Adjusted for Sex, Age, Temperature, Pulse, Respirations, SBP, History of diabetes, History of hyperlipidemia, ALB, TC, Cr, HCT, PLT

Regarding clinical outcomes, HRD-T1 showed the best prognosis, whereas LPI-T3 had the worst, with PMOF and mortality rates of 74.4% and 58.1% versus 30.8% and 18.4% in LGD-T2, and 30.4% and 14.3% in HRD-T1 (Table 4). Kaplan–Meier analysis confirmed significantly higher mortality risk in LPI-T3 (Log rank* P* < 0.05) (Fig. S5). Similar trajectory patterns and outcome distributions were validated in the MIMIC-IV cohort (Fig. S6 and Table S8). In further regression analysis, LPI-T3 was significantly associated with increased risk of in-hospital death, IPN, and PMOF compared with LGD-T2, while no significant difference was observed between HRD-T1 and LGD-T2. The HR for death in LPI-T3 was 3.00 (95% CI: 1.88–4.78), and the odds ratio for PMOF was 6.44 (95% CI: 2.79–14.84) (Fig. 8). In addition, subgroup analyses stratified by age, sex, and histories of hyperlipidemia and diabetes showed no significant interactions (all *P* for interaction > 0.05), and the direction of association remained consistent with the main findings (Fig. S7).

**Table 4:** Clinical outcomes of subjects grouped according to IAP Trajectories

	Total (n = 1,008)	HRD-T1 (n = 56)	LGD-T2 (n = 909)	LPI-T3 (n = 43)	*P*
LOS (days)	17.0 (11.0, 29.0)	17.5 (13.0, 37.0)	17.0 (11.0, 29.0)	18.0 (11.0, 29.0)	0.283
ANC, n (%)					0.185
No	464 (46.0)	25 (44.6)	425 (46.8)	14 (32.6)	
Yes	544 (54.0)	31 (55.4)	484 (53.2)	29 (67.4)	
IPN, n (%)					0.62
No	800 (79.4)	43 (76.8)	725 (79.8)	32 (74.4)	
Yes	208 (20.6)	13 (23.2)	184 (20.2)	11 (25.6)	
PMOF, n (%)					< 0.001
No	679 (67.4)	39 (69.6)	629 (69.2)	11 (25.6)	
Yes	329 (32.6)	17 (30.4)	280 (30.8)	32 (74.4)	
Death, n (%)					< 0.001
No	808 (80.2)	48 (85.7)	742 (81.6)	18 (41.9)	
Yes	200 (19.8)	8 (14.3)	167 (18.4)	25 (58.1)	

IAP, Intra-abdominal Pressure; LGD, Low-pressure Gradual Decline; HRD, High-pressure Rapid Decline; LPI, Low-pressure Progressive Increase; LOS, Length of hospital Stay; ANC, Acute Necrotic Collection; IPN, Infected Pancreatic Necrosis; PMOF, Persistent Multiple Organ Failure

## Discussion

This study is the first to elucidate the independent impact of both cumulative burden and dynamic change characteristics of IAP on the prognosis of SAP patients by integrating CumIAP and dynamic trajectory analysis. The results demonstrated a significant dose–response relationship between CumIAP and the risks of in-hospital death, PMOF, and IPN. Specifically, each SD increase in CumIAP elevated the in-hospital death risk by 37% and the PMOF risk by 86%. Notably, in this dataset, a distinctive U-shaped relationship between CumIAP and IPN was observed, with 79.44 mmHg representing an inflection point rather than a validated clinical cut-off. Mediation effect analysis further indicated that CumIAP partially mediated the effects of hypoalbuminemia and ANC, explaining up to 33.76% of ANC-related mortality. Additionally, LCGMM identified three distinct IAP dynamic trajectories (HRD-T1, LGD-T2, LPI-T3) with clear prognostic stratification. Significantly, although the HRD-T1 group had the highest baseline IAP, it showed the lowest PMOF incidence and mortality-likely attributable to its rapidly declining pressure pattern.

IAH, a prevalent complication in SAP patients, has well-established high incidence and strong prognostic associations. A prospective study by De Waele et al. [7] reported that 78% of SAP patients developed IAH (IAP ≥ 15 mmHg), with significantly higher incidences of respiratory, cardiovascular, and renal failure compared to non-IAH patients (all *P* < 0.001), consistent with our finding of a linear association between CumIAP and PMOF. Prior studies have also shown a dose-dependent relationship between IAH severity and mortality. Keskinen et al. found that SAP in-hospital mortality escalated with increasing IAP quartiles, reaching 50% in the highest quartile (25–33 mmHg) [9]. Similarly, Al-Bahrani et al. [30] demonstrated that IAH was associated with significantly higher median SOFA, APACHE II, and Multiorgan Dysfunction Scores, along with a 45% mortality rate. Rosas JM et al. [31] further confirmed a strong correlation between peak IAP and CT severity index (r = 0.62, *P* < 0.001). However, these studies were limited by reliance on single-point IAP measurements and did not capture the cumulative burden or dynamic changes in IAP, both of which may critically influence patient prognosis.

To overcome this limitation, this study calculated CumIAP during the first week of ICU admission in SAP patients using a time-weighted integration method, thereby quantifying the independent prognostic value of cumulative pressure burden. Each SD increase in CumIAP was associated with a 37% higher risk of in-hospital mortality, with risk rising progressively across CumIAP tertiles. The observed U-shaped relationship between CumIAP and IPN risk (*P* for nonlinearity = 0.004) may reflect a dual mechanism: moderately elevated IAP may limit necrosis spread via mechanical compression [32, 33], whereas excessive pressure impairs microcirculation and induces ischemia–reperfusion injury, increasing infection risk [34, 35]. In addition, ROC analysis showed that the AUC values of CumIAP for predicting mortality, IPN, and PMOF were 0.70, 0.67, and 0.69, respectively, indicating only acceptable discrimination and falling below the threshold typically considered strong (> 0.80). This suggests that CumIAP alone may not be sufficient as a robust predictive tool in clinical decision-making; rather, it may be more effective when integrated into multivariable prognostic models, and its predictive performance requires further validation in external datasets. Mediation analysis indicated that ALB levels negatively correlated with SAP poor prognosis, with CumIAP playing a negative mediating role, suggesting that higher ALB may improve outcomes partly by suppressing excessive CumIAP elevation, likely through maintaining intravascular colloid osmotic pressure and reducing fluid leakage [36, 37]. Conversely, CumIAP positively mediated ANC-related poor prognosis, as ANC-induced inflammatory occupation and exudate directly increase CumIAP, which in turn exacerbates pancreatic ischemia, disrupts the intestinal mucosal barrier, and promotes infection [38, 39], forming a vicious cycle that aggravates disease progression.

Dynamic trajectory analysis further illuminated the relationship between IAP fluctuations and SAP prognosis, aligning with Aitken et al.’s [40] longitudinal findings, which showed that non-survivors’ IAP progressively increased and survivors’ IAP stabilized after the third ICU Day. Using LCGMM, this study identified three optimal IAP trajectories: HRD-T1, LGD-T2, and LPI-T3. Compared with LGD-T2, the LPI-T3 group had a significantly higher mortality risk (HR = 3.0, 95% CI: 1.88–4.78), whereas HRD-T1 showed a non-significant 33% lower risk (HR = 0.67, 95% CI 0.32–1.38). Despite the HRD-T1 group having the highest baseline IAP (20.0 mmHg vs. 15.0 in LGD-T2 and 15.5 in LPI-T3), its mortality rate was the lowest (14.3% vs. 18.4% and 58.1%, respectively). The poor prognosis of the LPI-T3 trajectory may result from occult pancreatic necrosis progression, intestinal microcirculatory dysfunction with bacterial translocation [41, 42], and fluid overload-induced capillary leak syndrome [43], all contributing to sustained IAP elevation. In contrast, HRD-T1’s favorable outcomes likely stems from effective volume management, intestinal protection, early decompression, and inflammation control [44–47], enabling rapid IAP decline. These findings underscore the superiority of the IAP dynamic trajectory model over single-point measurements in capturing the dynamics of intra-abdominal hypertension and its clinical heterogeneity for prognosis.

This study offers an evidence-based framework for dynamic monitoring and stratified management of SAP. First, the identified U-shaped threshold between CumIAP and IPN risk may enable early identification of high-risk patients, and inform interventions such as restrictive fluid resuscitation combined with ALB supplementation to reduce secondary infection. Second, dynamic IAP trajectories outperform baseline measurements in prognostication, particularly by identifying patients with low initial IAP but rising trends. Third, trajectory-based classification can guide the timing of intervention: the progressive IAP increase and high mortality (58.1%) in the LPI-T3 group suggest a need for early minimally invasive drainage to halt IAH progression, whereas favorable outcomes in the HRD-T1 group support conservative management in patients with high baseline IAP but rapid decline. Integrating CumIAP burden and trajectory classification may enhance risk stratification, optimize treatment timing, and ultimately improve SAP outcomes. Moreover, based on a well-defined time-weighted integration method and LCGMM, the proposed CumIAP and trajectory models are highly programmable and readily integrable into electronic medical record systems or mobile applications. These tools enable automated calculation of CumIAP in SAP patients, identification of risk trajectories, comparison against critical thresholds, and generation of visual alerts-providing a reference for the digital and visualized management of SAP.

## Research advantages and limitations

This study’s strengths include: (1) a large sample size that enhances statistical power compared to previous studies; (2) the novel integration of CumIAP and IAP dynamic trajectory analysis to quantify their prognostic in SAP; (3) mediation analysis that highlights the role of IAP in SAP pathophysiology and provides new clinical and mechanistic insights.

However, some limitations should be acknowledged: (1) The retrospective design may introduce selection bias. Although robustness was checked through multiple imputation and sensitivity analysis, prospective validation is warranted. (2) The study population, limited to ICU-admitted SAP patients, which may reduce the generalizability to non-ICU populations or other ethnic groups. (3) IAP monitoring was restricted to the first 7 ICU days, potentially missing later pressure changes relevant to outcome. (4) External validation using the MIMIC-IV cohort was limited by a small sample size (n = 83) and incomplete IAP data, making it insufficient to fully verify key findings of this study—such as the U-shaped association between CumIAP and IPN and the identified risk threshold—which require confirmation in future prospective studies. (5) Despite adjusting for multiple variables—including medications and interventions such as surgery and PCD—in the regression and sensitivity analyses, the retrospective nature of this study may still lead to residual confounding.

## Conclusion

In conclusion, this study is the first to establish the prognostic significance of both CumIAP and IAP dynamic trajectories in SAP. CumIAP demonstrates linear positive correlations with in-hospital mortality and PMOF and a U-shaped relationship with IPN. Notably, patients with low baseline IAP but progressively increasing trends had worse outcomes than those with initially elevated but rapidly declining IAP. While these findings are promising, further prospective and multicenter validation is necessary to confirm the prognostic value of CumIAP and to establish standardized protocols for its clinical implementation.

## Supplementary Information

Supplementary file1 (DOCX 39817 KB)

Supplementary file 2 (DOCX 20 KB)

Supplementary file 3 (DOCX 43 KB)

## Data Availability

The data set used and/or analyzed during the current study available from the corresponding author reasonable request.

## Abbreviations

IAP: Intra-abdominal pressure
SAP: Severe acute pancreatitis
CumIAP: Cumulative IAP exposure
IPN: Infectious pancreatic necrosis
PMOF: Persistent multiple organ failure
ICU: Intensive care unit
IAH: Intra-abdominal hypertension
ACS: Abdominal compartment syndrome
APACHE II: Acute Physiology and Chronic Health Evaluation II
ALB: Albumin
ANC: Acute necrotic collection
LCGMM: Latent class growth mixture model
MIMIC-IV: Medical Information Mart for Intensive Care-IV
WBC: White blood cell count
NEU: Absolute neutrophil count
PLT: Platelet count
HCT: Hematocrit
TBIL: Total bilirubin
TG: Triglyceride
TC: Total cholesterol
Cr: Creatinine
BUN: Blood urea nitrogen
VFR: Volume of fluid resuscitation
PCD: Percutaneous catheter drainage
LOS: Length of hospital stay
VIF: Variance inflation factor
Conv: Convergence coefficient
PP: Posterior probability
SBP: Systolic blood pressure
DBP: Diastolic blood pressure
AUC: Area under the curve
