# Supplementary material for: Cumulative intra-abdominal pressure exposure and dynamic trajectories in ICU-admitted patients reveal prognostic determinants of severe acute pancreatitis
Source: World J Emerg Surg. 2025 Sep 29;20:74. doi: 10.1186/s13017-025-00646-y (PMC12481794; doi:10.1186/s13017-025-00646-y)
Supplement: Supplementary file 1 — Supplementary file1 (DOCX 39817 KB) [file 13017_2025_646_MOESM1_ESM.docx]

**Supplementary Figures**

**Supplementary Figure 1:** Q–Q Plots for Evaluating the Distribution of Continuous Covariates at Baseline.

**Supplementary Figure 2:** Schoenfeld Residual Plots Corresponding to In-hospital Death Over Time for CumIAP.

**Supplementary Figure 3:** ROC Analysis of CumIAP for Predicting In-Hospital Mortality, IPN, and PMOF.

**Supplementary Figure 4:** Stratified Analysis of the Association Between CumIAP and In-hospital Death. Adjusted Model: Adjusted for Sex, Age, Temperature, Pulse, Respirations, SBP, History of diabetes, History of hyperlipidemia, ALB, TC, Cr, HCT, excluding stratification variables.

**Supplementary Figure 5:** The Kaplan-Meier Survival Curves of Subjects Grouped by IAP Trajectory.

**Supplementary Figure 6:** Potential Class Change Trajectories of IAP in Subjects from the MIMIC Database. IAP: Intra-abdominal Pressure; LGD: Low-pressure Gradual Decline; HRD: High-pressure Rapid Decline; LPI: Low-pressure Progressive Increase.

**Supplementary Figure 7**: Subgroup Analysis of the Association between IAP Trajectory and the Risk of In-hospital Mortality in Subjects

Supplementary Figure 1: Q-Q Plots for Evaluating the Distribution of Continuous Covariates at Baseline.


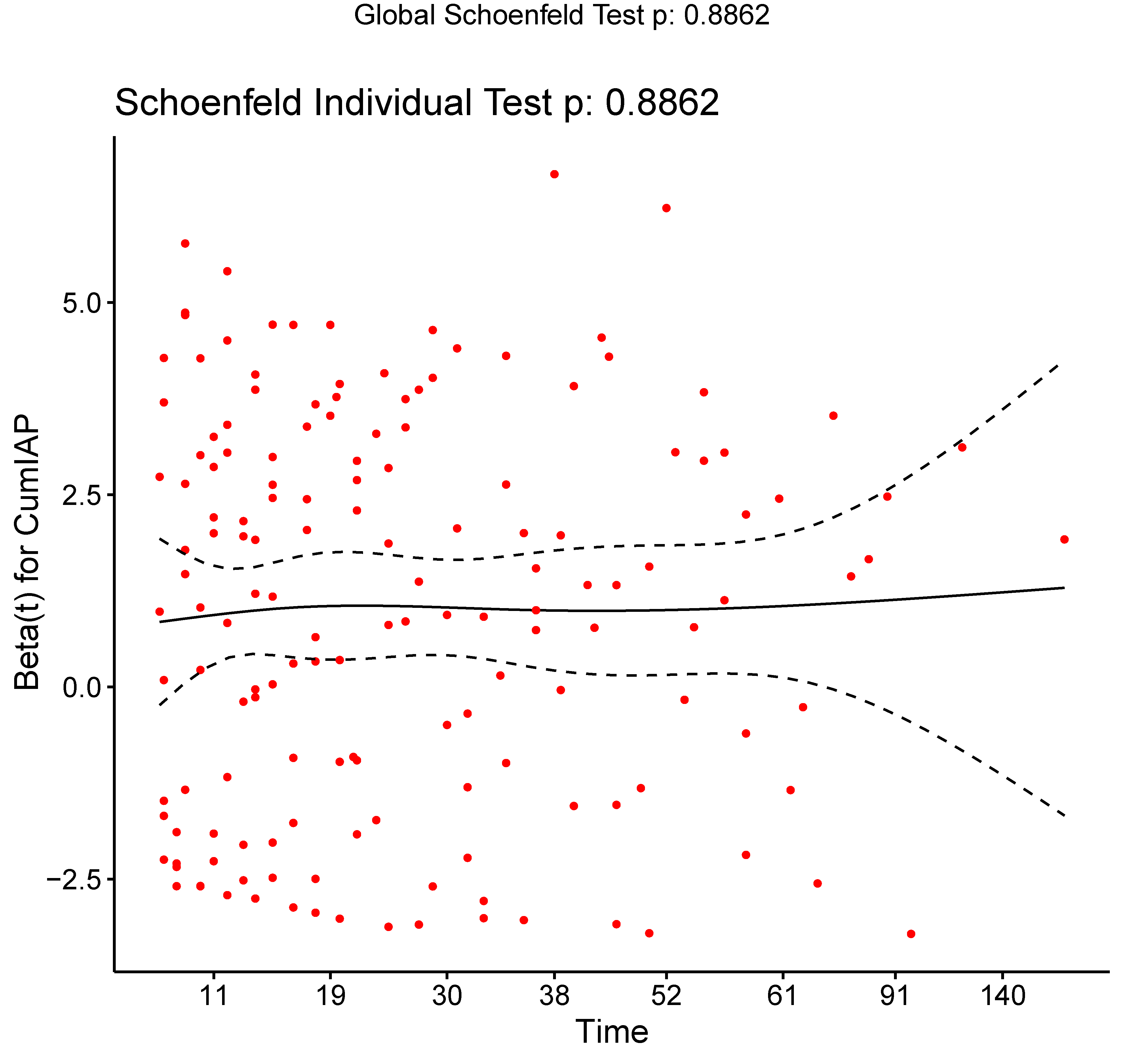
Supplementary Figure 2: Schoenfeld Residual Plots Corresponding to In-hospital Death Over Time for CumIAP


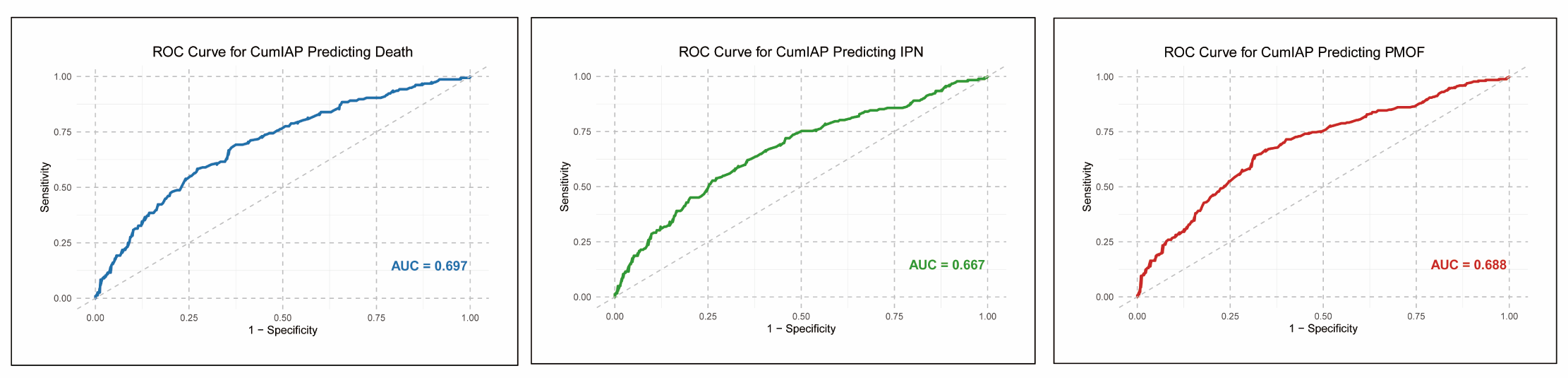


Supplementary Figure 3: ROC Analysis of CumIAP for Predicting In-Hospital Mortality, IPN, and PMOF


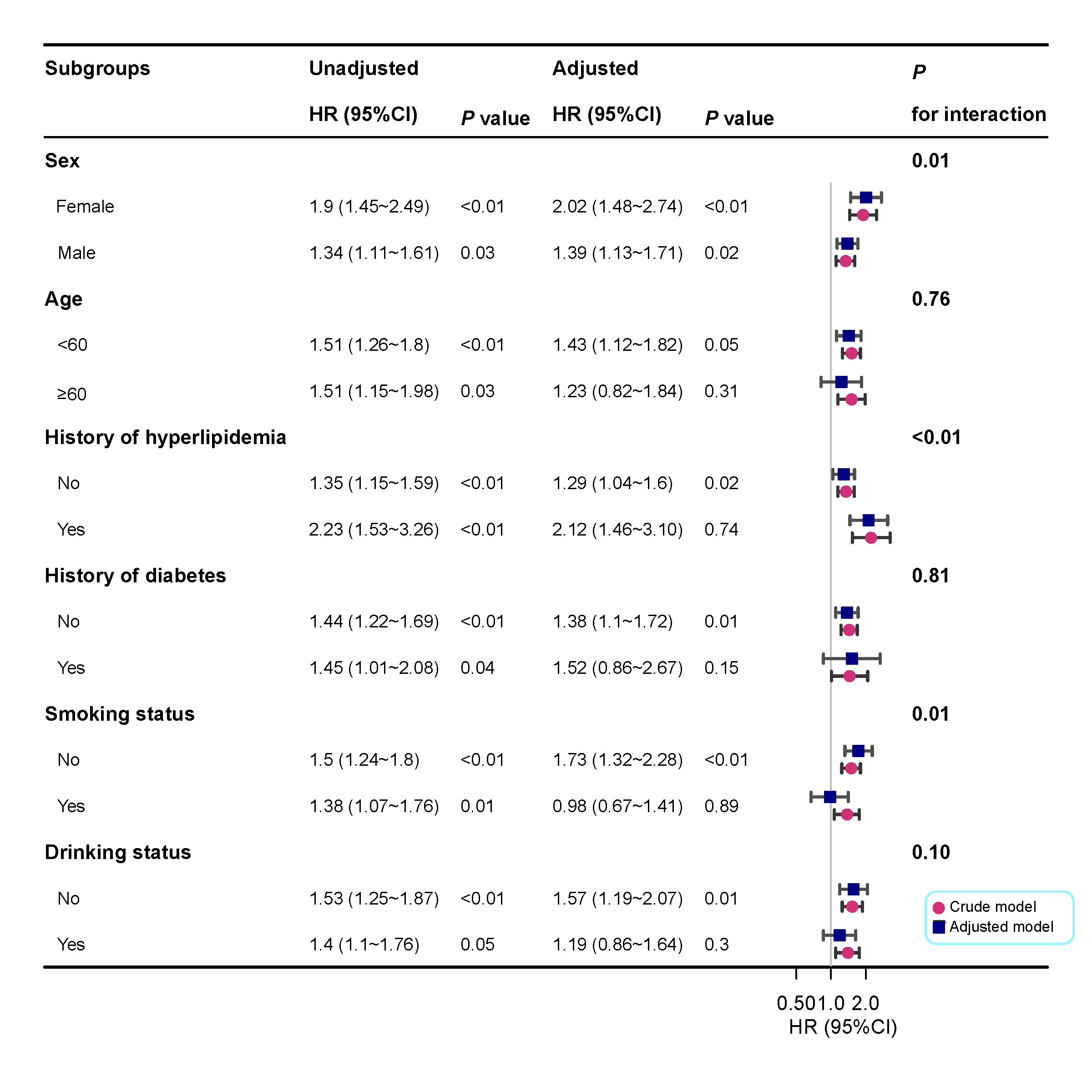


Supplementary Figure 4: Stratified Analysis of the Association Between CumIAP and In-hospital Death

Adjusted Model: Adjusted for Sex, Age, Temperature, Pulse, Respirations, SBP, History of diabetes, History of hyperlipidemia, ALB, TC, Cr, HCT, excluding stratification variables.


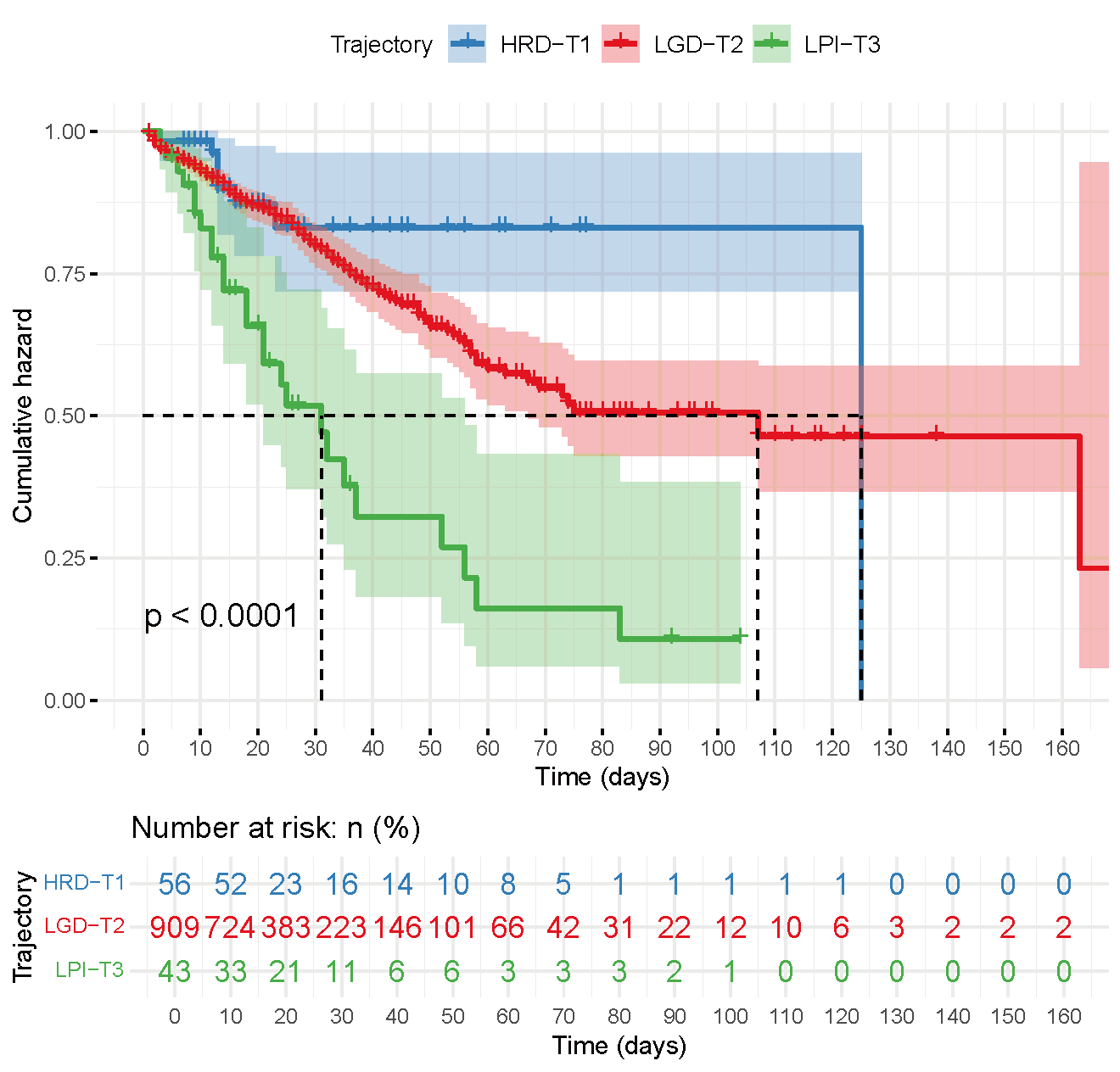


Supplementary Figure 5: The Kaplan-Meier Survival Curves of Subjects Grouped by IAP Trajectory


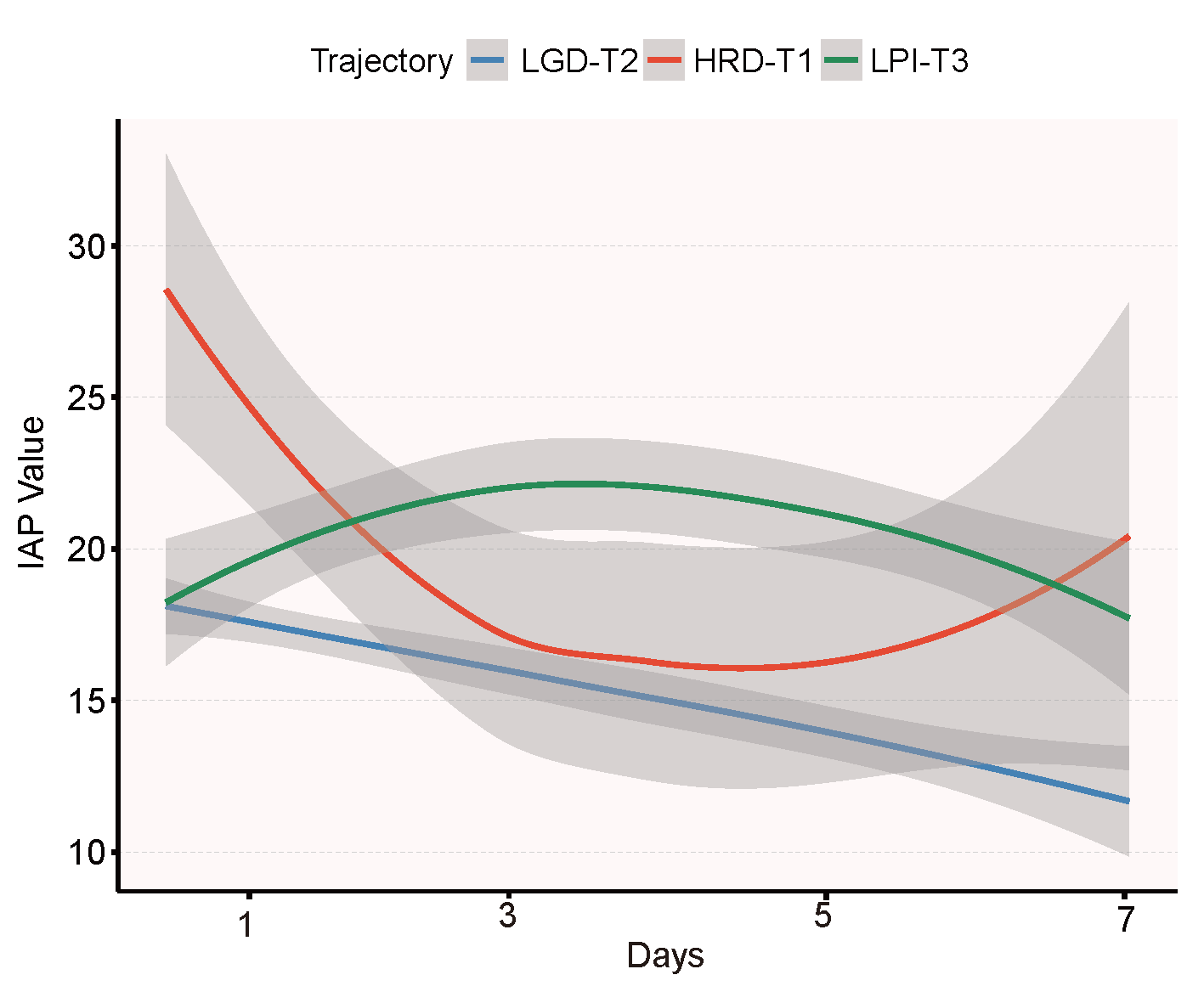


Supplementary Figure 6: Potential Class Change Trajectories of IAP in Subjects from the MIMIC Database. IAP: Intra-abdominal Pressure; LGD: Low-pressure Gradual Decline; HRD: High-pressure Rapid Decline; LPI: Low-pressure Progressive Increase.





Supplementary Figure 7: Subgroup Analysis of the Association between IAP Trajectory and the Risk of In-hospital Mortality in Subjects
