# Supplementary material for: Cumulative intra-abdominal pressure exposure and dynamic trajectories in ICU-admitted patients reveal prognostic determinants of severe acute pancreatitis
Source: World J Emerg Surg. 2025 Sep 29;20:74. doi: 10.1186/s13017-025-00646-y (PMC12481794; doi:10.1186/s13017-025-00646-y)
Supplement: Supplementary file 2 — (DOCX 20 KB) [file 13017_2025_646_MOESM2_ESM.docx]

**Supplementary Methods**

**Supplementary Method 1:** Study Design and Data Source

**Supplementary Method 2:** Ethical Approval

**Supplementary Method 3:** Handling of Missing Data

**Supplementary Method 4:** Sensitivity analysis

**Supplementary Method 5:** Analysis of Latent Class Growth Mixture Model (LCGMM)

**Supplementary Method 1: Study Design and Data Source**

This study is a retrospective cohort study that analyzed the patient data from the electronic medical database continuously maintained by the Department of Gastroenterology, the First Affiliated Hospital of Nanchang University (Jiangxi cohort, 2005-2023) and the Medical Information Mart for Intensive Care-IV database (MIMIC-VI v2.2, 2008-2019). Briefly, the Jiangxi cohort consecutively included inpatients diagnosed with acute pancreatitis (AP) in the Department of Gastroenterology, the First Affiliated Hospital of Nanchang University from 2005 to 2023. Professional clinicians entered data including patients' demographic characteristics, vital signs, main admission diagnoses, laboratory test indicators, in-hospital treatment conditions, and discharge outcomes. The MIMIC-IV 2.2 version is a free and open intensive care database that integrates data of over 50,000 ICU patients from Beth Israel Deaconess Medical Center during 2008-2019, covering various types of information such as demographic characteristics, vital signs, imaging examinations, laboratory test results, data dictionaries, and disease classification code files.

**Supplementary Method 2: Ethical Approval**

The research protocol of this study has been reviewed by the Institutional Review Board (IRB) of the First Affiliated Hospital of Nanchang University (No. 2011001). In addition, informed consent has been obtained from all subjects involved in the study. In addition, the MIMIC-VI v2.2 database has been approved by the IRB of the Massachusetts Institute of Technology, and the author (Shixuan Xiong) has obtained the access and extraction rights of the database (certification number: 58279303). All analysis processes and writing of the current study comply with the Declaration of Helsinki and the STROBE guidelines [17, 18].

**Supplementary Method 3: Handling of Missing Data**

For variables with missing values, the imputation method was determined according to the proportion of missing values of the variables [19, 20]. Specifically, the percentage of missing values of variables was first calculated (Supplementary table 1). It was found that the missing proportions of ALB (18.9275%) and TC (15.9463%) were relatively high, while those of Cr (2.9806%), HCT (2.6826%), and PLT (2.8316%) were relatively low. For variables with relatively high missing proportions, the K-nearest neighbors (Knn) imputation method was used for processing; for other variables with relatively low missing proportions, the mean imputation method was applied.

**Supplementary Method 4: Sensitivity analysis**

Sensitivity analysis 1: The correlation analysis between CumIAP and the risk of in-hospital death of the subjects was repeated in the imputed dataset.

Sensitivity analysis 2: The adjusted four baseline vital signs (body temperature, pulse, respiration, SBP) were replaced with the 7-day cumulative exposure values calculated by the corresponding indicators.

Sensitivity analysis 3: Additional adjustments were made for whether the patients used ALB, low molecular weight heparin, and insulin.

Sensitivity analysis 4: Subjects with a relatively high risk of death (Age ≥ 75 years old) were further excluded.

Sensitivity analysis 5: Additional adjustments were made for the PCD, Surgical, and CumVFR based on fully adjusted model.

**Supplementary Method 5: Analysis of Latent Class Growth Mixture Model (LCGMM)**

To supplement the dynamic fluctuation information lacking in CumIAP, LCGMM analysis was carried out on the subjects who had at least two IAP measurement values within 7 days of ICU admission. LCGMM is a statistical model for analyzing longitudinal data. It combines the characteristics of latent class analysis and growth model, allows for random effects within classes, and can identify different latent classes in the data while considering the individual trends over time, with each class having a unique growth trajectory. The change pattern of IAP within 7 days after the patients' ICU admission was fitted by maximum likelihood estimation. To fit the optimal trajectory, an LCGMM containing 2-5 classes was constructed using a cubic polynomial function. The model was selected based on the principles of the convergence coefficient Conv (1: convergence; 2: non-convergence), minimization of the Bayesian Information Criterion (BIC), maximization of the entropy value (Entropy), and a posterior probability (PP) > 70% [26-29]. Subsequently, the baseline characteristics and various clinical prognosis indicators of the subjects were described and compared according to the IAP trajectory groups, and the Kaplan-Meier curves were used to describe the cumulative survival risks of the subjects in each trajectory group. The IAP trajectory groups were included in the above regression model as categorical variables to analyze their association with the poor prognosis of the subjects. In addition, the data of SAP patients admitted to the ICU in the MIMIC-VI database (n = 83) were used to repeat the construction of the trajectory model and compare the distribution and prognosis of each group of people.
