# Supplementary material for: Cumulative intra-abdominal pressure exposure and dynamic trajectories in ICU-admitted patients reveal prognostic determinants of severe acute pancreatitis
Source: World J Emerg Surg. 2025 Sep 29;20:74. doi: 10.1186/s13017-025-00646-y (PMC12481794; doi:10.1186/s13017-025-00646-y)
Supplement: Supplementary file 3 — (DOCX 43 KB) [file 13017_2025_646_MOESM3_ESM.docx]

**Supplementary Tables**

**Supplementary Table 1:** Proportion of Missing Data per Variable in the Final Regression Model.

**Supplementary Table 2:** Checking the Normality of Baseline Continuous Covariates Using the Kolmogorov-Smirnov Test.

**Supplementary Table 3:** Collinearity Diagnostics of the Associations Between CumIAP and In-hospital death, IPN, PMOF.

**Supplementary Table 4:** ROC Analysis of CumIAP for Identifying Death, IPN, and PMOF.

**Supplementary Table 5:** Sensitivity Analysis-1: Correlation Analysis between CumIAP and In-hospital Death before and after Imputation of Missing Data.

**Supplementary Table 6:** Sensitivity Analysis 2-5: Correlation Analysis between CumIAP and In-hospital Death.

**Supplementary Table 7:** Statistics for Choosing the Best Number of Trajectories for Jiangxi cohort.

**Supplementary Table 8:** Outcomes of Subjects Grouped by IAP Trajectories in the MIMIC Database.

Supplementary Table 1: Proportion of Missing Data per Variable in the Final Regression Model

| Variable | Missing frequent | Missing percentage % |
| --- | --- | --- |
| Age | 0 | 0 |
| ALB | 127 | 18.9275 |
| SBP | 0 | 0 |
| Cr | 20 | 2.9806 |
| CumIAP | 0 | 0 |
| Death | 0 | 0 |
| HCT | 18 | 2.6826 |
| History of diabetes | 0 | 0 |
| History of hyperlipidemia | 0 | 0 |
| PLT | 19 | 2.8316 |
| Pulse | 0 | 0 |
| Respirations | 0 | 0 |
| Sex | 0 | 0 |
| TC | 107 | 15.9463 |
| Temperature | 0 | 0 |

Abbreviations as in Table 1.

Supplementary Table 2: Checking the Normality of Baseline Continuous Covariates Using the Kolmogorov-Smirnov Test

| **Variables** | **Statistic** | ***P* value** |
| --- | --- | --- |
| age | 0.0555 | <0.001 |
| weight | 0.0970 | <0.001 |
| Temperature | 0.1109 | <0.001 |
| Pulse | 0.0413 | <0.001 |
| Respiration | 0.1054 | <0.001 |
| SBP | 0.0367 | 0.002798 |
| DBP | 0.0366 | 0.002853 |
| IAP | 0.1361 | <0.001 |
| TBIL | 0.2311 | <0.001 |
| Cr | 0.2221 | <0.001 |
| CRP | 0.1005 | <0.001 |
| ALB | 0.1039 | <0.001 |
| TC | 0.1984 | <0.001 |
| TG | 0.2337 | <0.001 |
| APACHE II | 0.0580 | <0.001 |
| BUN | 0.1610 | <0.001 |
| WBC | 0.0853 | <0.001 |
| PLT | 0.0698 | <0.001 |
| HCT | 0.0372 | 0.002194 |
| VFR | 0.1181 | <0.001 |

Abbreviations: IAP: Intra-abdominal Pressure; SBP: Systolic Blood Pressure; DBP: Diastolic Blood Pressure; TBIL: Total Bilirubin; Cr: Creatinine; CRP: C-Reactive Protein; ALB: Albumin; TC: Total Cholesterol; TG: Triglyceride; APACHEII: Acute Physiology and Chronic Health Evaluation II; BUN: Blood Urea Nitrogen; WBC: White Blood Cell; NEU: Neutrophil; PLT: Platelet; HCT: Hematocrit; VFR: Volume of Fluid Resuscitation.

Supplementary Table 3: Collinearity Diagnostics of the Associations Between CumIAP and In-hospital death, IPN, PMOF

|  | Variance Inflation Factor | | |
| --- | --- | --- | --- |
|  | Death | IPN | PMOF |
| Variables | Step 1 | Step 1 | Step 1 |
| CumIAP | 1.1 | 1.1 | 1.1 |
| Sex | 2 | 2 | 2 |
| Age | 1.5 | 1.5 | 1.5 |
| Smoking status | 1.8 | 1.8 | 1.8 |
| Drinking status | 1.7 | 1.7 | 1.7 |
| History of hyperlipidemia | 1.2 | 1.2 | 1.2 |
| History of diabetes | 1.2 | 1.2 | 1.2 |
| Temperature | 1.2 | 1.2 | 1.2 |
| Pulse | 1.5 | 1.5 | 1.5 |
| Respirations | 1.3 | 1.3 | 1.3 |
| SBP | 2.7 | 2.7 | 2.7 |
| DBP | 2.6 | 2.6 | 2.6 |
| WBC | 1.2 | 1.2 | 1.2 |
| HCT | 1.3 | 1.3 | 1.3 |
| PLT | 1.2 | 1.2 | 1.2 |
| AST | 1.1 | 1.1 | 1.1 |
| TG | 3.2 | 3.2 | 3.2 |
| TC | 3.2 | 3.2 | 3.2 |
| Cr | 1.2 | 1.2 | 1.2 |

Note-1: Variance inflation factor = 1/(1-R^2^). Abbreviations as in Table 1.

Note-2: The variables with variance inflation factor >5 will be regarded as collinear variables and cannot be included in the multiple regression model.

Supplementary Table 4: ROC Analysis of CumIAP for Identifying Death, IPN, and PMOF

| Outcomes | Yes | No | AUC | 95%CI low | 95%CI up | Best threshold | Specificity | Sensitivity |
| --- | --- | --- | --- | --- | --- | --- | --- | --- |
| Death | 156 | 515 | 0.70 | 0.65 | 0.74 | 90.91 | 0.63 | 0.69 |
| IPN | 184 | 487 | 0.67 | 0.62 | 0.71 | 89.25 | 0.73 | 0.54 |
| PMOF | 274 | 397 | 0.69 | 0.65 | 0.73 | 90.91 | 0.69 | 0.64 |

Abbreviations: AUC: Area Under the ROC Curve; CI: Confidence Interval; IPN: Infected Necrotizing Pancreatitis; PMOF: Persistent Multiple Organ Failure.

Supplementary Table 5: Sensitivity Analysis-1: Correlation Analysis between CumIAP and In-hospital Death before and after Imputation of Missing Data

|  |  |  | Before imputation | | After imputation | |
| --- | --- | --- | --- | --- | --- | --- |
| Variable | Crude  HR (95%CI) | *P* value | Adjusted HR (95%CI) | *P* value | Adjusted HR (95%CI) | *P* value |
| CumIAP (per SD) | 1.44 (1.25~1.67) | <0.001 | 1.37 (1.12~1.67) | 0.002 | 1.45 (1.23~1.71) | <0.001 |
| CumIAP tertiles | |  |  |  |  |  |
| Tertile 1 | 1(Ref) |  | 1(Ref) |  | 1(Ref) |  |
| Tertile 2 | 1.23 (0.75~2.02) | 0.411 | 1.36 (0.76~2.43) | 0.325 | 1.23 (0.74~2.07) | 0.424 |
| Tertile 3 | 2.28 (1.46~3.57) | <0.001 | 2.36 (1.35~4.1) | 0.002 | 2.34 (1.45~3.78) | 0.001 |

HR: Hazard ratio; CI: Confidence interval; SD: Standard deviation; Other abbreviations as in Table 1.

Crede Model was adjusted for: None.

Adjusted Model: Adjusted for Sex, Age, Temperature, Pulse, Respirations, SBP, History of diabetes, History of hyperlipidemia, ALB, TC, Cr, HCT, PLT.

Supplementary Table 6: Sensitivity Analysis 2-4: Correlation Analysis between CumIAP and In-hospital Death

|  |  | Sensitivety-2 | Sensitivety-3 | Sensitivety-4 | Sensitivety-5 |
| --- | --- | --- | --- | --- | --- |
| Variable | Crude HR  (95%CI) | Adjusted HR (95%CI) | Adjusted HR (95%CI) | Adjusted HR (95%CI) | Adjusted HR (95%CI) |
| CumIAP (Per SD) | 1.44 (1.25~1.67) | 1.28 (1.05~1.58) | 1.41 (1.15~1.74) | 1.39  (1.13, 1.71) | 1.33  (1.08，1.64) |
| CumIAP Tertiles |  |  |  |  |  |
| Tertile1 | 1(Ref) | 1(Ref) | 1(Ref) | 1(Ref) | 1(Ref) |
| Tertile2 | 1.23 (0.75~2.02) | 1.39 (0.77~2.51) | 1.29 (0.72~2.31) | 1.28  (0.70, 2.35) | 1.34 (0.75~2.4) |
| Tertile3 | 2.28 (1.46~3.57) | 2.26 (1.28~3.99) | 2.34 (1.35~4.05) | 2.37  (1.35, 4.19) | 2.29 (1.28~4.08) |

Abbreviations: HR: Hazard ratio; CI: Confidence interval; SD: Standard deviation; Other abbreviations as in Table 1.

Crede Model was adjusted for: None.

Sensitivety-2: In the fully adjusted model, replace the four major vital signs in the regression model with time-series variables of cumulative exposure (calculate their cumulative exposure values).

Sensitivety-3: In the fully adjusted model, additional adjustments were made for the use of low-molecular-weight heparin, albumin, and insulin.

Sensitivety-4: Subjects older than 75 years of age were excluded.

Sensitivety-5**：**In the fully adjusted model, additional adjustments were made for the PCD, Surgical, and CumVFR.

Supplementary Table 7: Statistics for Choosing the Best Number of Trajectories for Jiangxi cohort.

| G | Conv | BIC | SABIC | entropy | PP1 | PP2 | PP3 | PP4 | PP5 |
| --- | --- | --- | --- | --- | --- | --- | --- | --- | --- |
| 2 | 1 | 22863.63 | 22812.81 | 0.005894 | 0.5345 | 0.536 | - | - | - |
| 3 | 1 | 22859.88 | 22796.36 | 0.737988 | 0.7794 | 0.9117 | 0.7412 | - | - |
| 4 | 1 | 22881.66 | 22805.44 | 0.569773 | 0.7318 | 0.7705 | 0.768 | 0.633 | - |
| 5 | 1 | 22894.05 | 22805.12 | 0.595424 | 0.7211 | 0.8004 | 0.579 | 0.7514 | 0.64 |

Abbreviations: BIC, Bayesian information criteria; SABIC, sample-adjusted information criteria; PP, posterior probability.

Supplementary Table 8: Outcomes of Subjects Grouped by IAP Trajectories in the MIMIC Database

| Variables | Total  (n = 83) | HRD-T1  (n = 7) | LGD-T2  (n = 62) | LPI-T3  (n = 14) | *P* | |
| --- | --- | --- | --- | --- | --- | --- |
| Is dead, n (%) | |  |  |  | 0.637 | |
| No | 61 (73.5) | 6 (85.7) | 46 (74.2) | 9 (64.3) |  | |
| Yes | 22 (26.5) | 1 (14.3) | 16 (25.8) | 5 (35.7) |  | |
| Is hospital dead, n (%) | |  |  |  | 0.348 | |
| No | 69 (83.1) | 6 (85.7) | 53 (85.5) | 10 (71.4) |  | |
| Yes | 14 (16.9) | 1 (14.3) | 9 (14.5) | 4 (28.6) |  | |
| Is icu dead, n (%) | |  |  |  | 0.066 | |
| No | 73 (88.0) | 6 (85.7) | 57 (91.9) | 10 (71.4) |  | |
| Yes | 10 (12.0) | 1 (14.3) | 5 (8.1) | 4 (28.6) |  | |
| Death within hospital 28 days, n (%) | | | |  | | 0.22 |
| No | 71 (85.5) | 6 (85.7) | 55 (88.7) | 10 (71.4) |  | |
| Yes | 12 (14.5) | 1 (14.3) | 7 (11.3) | 4 (28.6) |  | |
| Death within icu 28days, n (%) | | | |  | | 0.215 |
| No | 71 (85.5) | 6 (85.7) | 55 (88.7) | 10 (71.4) |  | |
| Yes | 12 (14.5) | 1 (14.3) | 7 (11.3) | 4 (28.6) |  | |

Abbreviations: IAP: Intra-abdominal Pressure; LGD: Low-pressure Gradual Decline; HRD: High-pressure Rapid Decline; LPI: Low-pressure Progressive Increase; ICU: Intensive Care Unit.
